# Indents

**Published:** 1914-03-15

**Authors:** 


### INDENTS.

☞Brief Articles of Technical or Scientific Value will receive notice and comment. Contributions invited.

*Dental Mutilation by Savages.* A missionary of the White Nile district, says that the Jieng Negroes, for some traditional reason which they can't explain have six of their lower front teeth extracted. Although they are great dandies this mutilation can't be for esthetic reasons. They clean their teeth with a native made tooth brush and wood ashes.—*British Dental Journal.*

*Hygienic Care of the Toothbrush.* Dr. Funcke, in the *Deutsche Monatschrift,* gives the following as a result of some experiments he made to secure clean and steril tooth brushes after using. He found that 70 percent. alcohol solution in water was most effective, but that it dissolved some forms of brush handles and colored the metal wires used in fastening the bristles into the handles. He found hard rubber handles, silk thread and phosphurated wire the best means of attaching the bristles. He advocates suspending the brush into a wide-mouth glass bottle or jar, containing the alcoholic solution, by fastening the handle free end of the brush into a rubber cork or similar means of stoppering the bottle, so as to keep the solution from evaporating. He noted that brushes kept in the alcohol solution were not only sterilized but they lasted longer. The solution should be thrown out at least every two weeks, and care should be taken that by the frequent dipping of wet brushes into it that its alcoholic content did not fall below 55 per cent. of alcohol. The brushes should of course be thoroughly washed and rinsed in clean water after each using. He found that solutions containing phydrogen dioxygen, or formalin should not be used.

*Alcoholic Drinks Favor Dental Caries.* Dr. Floxas reports as a result of examination of Mohammedan workmen who were not total abstainers that dental caries increased from the ages of 20 to 50 years from approximately 2 per cent. to 17 per cent. He does not claim that alcohol is the only or main cause of carious teeth, but that it seems to be a significant attendant, as the tetotalers ranged from one half per cent. at 20 years, to only four and one-half per cent. at 50 years.

*Broader Conceptions of Technical Operations.* As primary conceptions, around which my thoughts have gathered, I desire to call to your attention the fact that for generations our profession has too often been content to make good bridges, crowns, fillings and inlays without sufficient consideration of their relation to the masticating organ as a whole; that we have too often exercised our ingenuity to retain individual teeth incapable of restoration to efficiency or sanitary condition long after they have become a menace to the life and health of the owners; that we have too often focused on the tooth and too seldom on the organ of which it is a part; and that the post-operative details are the ones now most in need of practical solution in the cure and maintenance of cure in advance pyorrhea.

The elimination of vicious occlusal stress, the establishment of conditions of mutual support for weakened teeth, provision for maintenance of cleanliness by the patient, and efficient functioning of the masticating organ, are essential features of the cure of advanced pyorrhea, and the pyorrhea specialist who looks for permanent success must find a way to deal with these factors.—*Dr. Gillett in January, 1914, Summary.*

*Importance of Mutilated Occlusion.* No argument is needed in support of the statement that every case presenting to the dentist with a mutilated masticating organ must be considered a grave one, and that its presentation imposes upon him the imperative duty of a careful study of the masticating organ as a unit before he determines his procedure with regard to an individual member of that organ.—*Dr. Gillett, in Summary.*

*Cause of the Breaking of Facings From Backings.* The breaking of platinum pin facings from backings is commonly, but wrongly, attributed to either physical or mechanical factors. It is due to chemical phenomena taking place in soldering the facing and backing together, but especially to the action of heat. The breaking of platinum pins is most commonly brought about by an agent which has transformed the hard and fibrous metal into a crystalline and fragile one. Though neither air nor heat induces oxidation of platinum, and platinum is not attacked by any acid, there are nevertheless certain substances with which it unites at a relatively low temperature, and it is specially the contact with easily-reducible metals that endangers the pins. Certain solders and even some investing materials contain base metals, such as iron, antimony, tin, and zinc, which in uniting with platinum under the blowpipe bring about its fragility. Carbon is another agent which attacks platinum, and this cause of the trouble is the more important, as many sorts of wax not rarely contain resin or other impurities which, by the heat of the blowpipe, are transformed into carbon.—*Laboratoire et Progress Dentaire, per Dental Surgeon.*

*Alveolar Abscess With Fatal Termination.* A case recently treated at the Kent and Canterbury Hospital of alveolar abscess arising from a deciduous tooth, followed by general septicemia and subsequent death, is of exceptional interest owing to the grave sequelae proceeding from an apparently trivial and commonplace ailment.

F. M., age nine, son of a laborer, attended the dental department of the hospital. His left cheek was swollen, and on the buccal surface of the gum over the root of the lower second deciduous molar, which was carious, was a small gumboil.

His general appearance was that of a weakly child, but his mouth did not seem to be in a more unhealthy or unclean condition than is usually found in children of his class.

The tooth was easily removed, and on leaving the hospital he was instructed to rinse his mouth frequently with hot water during the next day or two. The forceps used for the extraction were sterilized previously. The following are the notes of the house surgeon:

On admission to the hospital the patient was unconscious, temperature being 102° F., troublesome cough and very foul breath, left cheek swollen and painful, and jaw rigid. On examination moist rales and impaired resonance over lower lobe of right lung. Though the local condition of the jaw cleared up in two days under formentations, the general condition grew progressively worse, and symptoms of meningitis supervened. The child died. Post-mortem revealed septic pneumonia of right lung, principally lower lobe, and purulent basal meningitis.—*R. S. N. Faro, British Dental Journal.*

*Metal Spraying Process.* A German Engineer by the name of Schoop has invented a process of volatiling melted metal and spraying them over metal and other surfaces by means of a high pressure gas so that a more or less smooth and thick deposit of the metal is obtained which is held in place durably by the conditions under which it is done. Dr. Lichtwitz in the *Deutsche Monatschrift* for April, 1913, thinks the invention has an important dental application.

The thickness and durability of the deposits is under control, and it is suggested that metal dentures can be constructed by coating a plaster model with a metal deposit of sufficient thickness to ensure tensil strength, making the plate and teeth attachment all in one piece. He also suggests filling cavities in natural teeth by depositing finely divided gold or other metal in the cavity. This would result in accurate work and in case it can be made practicable it will undoubtedly put out of business all our present methods.

Several years ago some one conceived the idea of depositing gold by electralysis in tooth cavities, but it never materialized. If we can blow our tooth cavities full of some insoluable material, the day of gold, amalgam and cemented fillings will speedily pass away and some of our dentists will become farmers or automobile drivers.

*Automobile as a Cause of Caries.* According to a statement in the British Dental Journal the drivers of the automobile omnibus on the crowded London streets are specially prone to caries of the teeth. The fact is noticeable by the employers as well as the men. The explanation given is that the nerve strain of stopping and starting the cars and in dodging people and other vehicles is so constant and demands such alert vigilence that the whole nervous system becomes excited or irritated, and as a consequence the oral secretions become vitiated and so lose their antiseptic power and function. It is said that on certain routes a driver will stop 480 times in a four hour shift, which would involve several thousand actual movement of levers, etc., with the hands and feet in addition to the mental aggitation accompanying stops under perilous circumstances. We shall soon have a new word in our nomenclature, how would odontomobileitis do?

*Alopecia of Dental Origin.* Dr. Jacquet of Paris, France, believes that alopecia has a reflex rather than a microbic origin, and reports to the French Academy of Medicine a case substantiating his theory. He reports the case of a lady suffering from severe periodontitis who experienced sever pain in the left parietal region every time the tooth was disturbed, and always at the same spot. The tooth was extracted and almost immediately a patch of alopecia appeared at the exact spot where the referred pain had been experienced. The alopecia, however, did not continue, but made a rapid and permanent recovery in a short time after the tooth was extracted.—*British Dental Journal, January 1914.*

*True Recreation.* We hear much of the need of relaxation for the "tired business man" and the "jaded society woman." Their needs are supposed to be met, in the first place, by amusements devised for inert and vacant minds, and, in the last resort, by the "rest-cure." Meanwhile as a recent writer[1] observes, our octogenarian colleague whose name is so frequently associated with this therapeutic measure is demonstrating in his own person the fact that one may fill well-ordered days with steady accomplishment and find recreation, not in passivity, but in a complete change of mental occupation. Thirty years ago Dr. S. Weir Mitchell undertook to write a novel as a vacation pastime after his winter practice. The result was "In War Time," which had a large and immediate success. The effect on Dr. Mitchell himself, even before the success of his novel came to corroborate it, was a thorough recreation, a veritable refreshment of faculties that sent him back to his work reinvigorated and satisfied. He has continued since to take up some serious literary labor every summer. His vigor of mind and body now, when well past four score years of age, attest, the healthfulness of this practice begun in middle life. It is possible, indeed, that rather too exclusive stress is laid on it in the article mentioned. Doubtless Dr. Mitchell's health and long life are due in large measure to his habit of changing his environment as well as his occupation every summer. At all events, his example is instructive. It is well to remember that many patients whose nervous condition is on the down grade toward nervous exhaustion need, not rest or frivolous amusement, but a new and absorbing mental occupation that will take their minds entirely out of the beaten tracks of their previous interests.—*Journal A. M. A.*

1Versatility and Dr. S. Weir Mitchell, Century Magazine, December, 913.

